# Antibiotic exposure and recovery of persister states influence virulence phenotypes in *Pseudomonas aeruginosa*

**DOI:** 10.1007/s00203-026-04995-3

**Published:** 2026-06-16

**Authors:** Waleska Stephanie da Cruz Nizer, Carlos Eduardo Dias Igídio, Estela Mafra Ribeiro, Allanis Cristiny Oliveira Andrade, Samantha Neves de Oliveira, Mauro Martins Teixeira, Daniele da Glória de Souza, Caio Tavares Fagundes

**Affiliations:** 1 Host-Microorganism Interaction Laboratory, Department of Microbiology, Institute of Biological Sciences, Federal University of Minas Gerais, Belo Horizonte, MG Brazil; 2 Drug Research and Development Center, Institute of Biological Sciences, Federal University of Minas Gerais, Belo Horizonte, MG Brazil

**Keywords:** *Pseudomonas aeruginosa*, Gram-negative bacteria, β-lactams, Fluoroquinolone, Persistence, Biofilms

## Abstract

The pathogenicity of *P. aeruginosa* is not only mediated by its resistance mechanisms but also by its ability to form persister cells. The persister state is a transient condition in which cells retain a dormant, non- or slow-growing state during exposure to stressors, such as high doses of bactericidal antibiotics, but can resume growth once the stress is removed. In this study, we investigated in vitro the virulence phenotype and stress-induced modulation of persisters in *P. aeruginosa* PA14 exposed to 30 × the minimum inhibitory concentration (MIC) of imipenem (60 µg/mL) or ciprofloxacin (3.75 µg/mL). Using a biphasic killing assay, we detected persisters with survival levels of 4.8 and 3.4 log_10_ CFU/mL at 24 h for imipenem and ciprofloxacin, respectively. MIC assays confirmed the absence of acquired resistance. Flow cytometry revealed a time-dependent accumulation of cells with intermediate redox activity and membrane integrity, consistent with the persister phenotype. Both planktonic and biofilm-associated populations harbored persisters, with biofilms exhibiting a greater amount of tolerant cells. Furthermore, persister cells displayed reduced pigment production, biofilm formation, and phagocytosis survival. Gene expression analysis revealed upregulation of *higB* and *pqsA* in persisters, along with distinct profiles in recovered cells. Pre-exposure to hydrogen peroxide (H_2_O_2_; 0.4%) increased survival by > 1 log_10_ following antibiotic treatment. Additionally, pretreatment with 5-fluorouracil (5-FU; 256 µg/mL) increased imipenem persistence by twofold. Altogether, our findings show that oxidative and genotoxic stresses promote persistence, and that macrophage interactions modulate persister physiology, offering insight into host–pathogen dynamics and potential therapeutic avenues.

**Supplementary Information:**

The online version contains supplementary material available at https://doi.org/10.1007/s00203-026-04995-3.

## Introduction

Persistence is a phenomenon in which a subpopulation of bacterial cells survives various stress conditions, such as nutrient starvation, oxidative stress, and exposure to antimicrobial agents. Persister cells display a transient state of dormancy or a state of markedly reduced metabolic activity (Balaban et al. 2019). This adaptive strategy was first reported in 1942 by Gladys Hobby and colleagues, who observed that approximately 1% of *Pneumococcus*, hemolytic *Streptococcus*, and *Staphylococcus* populations survived penicillin treatment (Hobby et al. 1942). The term “persisters” was later introduced by Joseph Bigger to describe these surviving, non-resistant cells (Bigger 1944). Unlike antibiotic-resistant bacteria, which can grow and divide in the presence of antimicrobials, persister cells remain non-replicative and metabolically inactive during treatment. However, once the stress is removed, the population can resume normal growth (Balaban et al. 2019).

Detection of persister cells has been documented in a wide range of bacterial species, including *Staphylococcus aureus* (Conlon et al. 2016; Peyrusson et al. 2020), *Escherichia coli* (Vázquez-Laslop et al. 2006), *Mycobacterium tuberculosis* (Zhang et al. 2012; Wang et al. 2024), *Pseudomonas aeruginosa* (Zadeh et al. 2022; Patel et al. 2022; Roy et al. 2024), and *Salmonella* spp. (Cheverton et al. 2016; Drescher et al. 2019). Among these, *P. aeruginosa* stands out as one of the most clinically relevant pathogens associated with persistent infections (Mulcahy et al. 2010; La Rosa et al. 2025). This Gram-negative opportunistic pathogen is responsible for severe, difficult-to-eradicate infections, such as ventilator-associated pneumonia and burn and chronic wound infections, particularly in immunocompromised individuals. In addition to its resistance to diverse classes of antimicrobials, *P. aeruginosa* exhibits high metabolic adaptability and a remarkable ability to form biofilms (Bassetti et al. 2018; Qin et al. 2022). The emergence of persistence further enhances its pathogenicity, allowing a subpopulation of cells to survive antibiotic therapy and driving infection recurrence and chronicity. For instance, in patients with cystic fibrosis (CF), in which *P. aeruginosa* colonization occurs in 60–70% of cases (Crull et al. 2018), persister cells play a pivotal role in the establishment and long-term maintenance of chronic pulmonary infections (Malhotra et al. 2019). In this context, Bartell et al. (2020) reported that approximately 19% of *P. aeruginosa* isolates from CF airways exhibited a persister phenotype (Bartell et al. 2020).

While some mechanisms have already been implicated in persister cell phenotype (Pan et al. 2023), this phenomenon is considered a multifactorial process (Maisonneuve et al. 2018; Bartell et al. 2020). Among the most studied mechanisms are the toxin-antitoxin (TA) systems and the stringent and SOS responses (Pan et al. 2023). Under stress conditions, the antitoxin is degraded by cellular proteases, leading to toxin activation, which can then inhibit essential cellular processes, such as DNA replication, transcription, and protein synthesis (Yang and Walsh 2017). One well-characterized protease involved in this process is Lon (Kamruzzaman et al. 2021). In *E. coli*, overexpression of the toxins RelE, which inhibits translation, and HipA, a toxin of the *hipAB* TA system, has been associated with increased persister cell detection (Keren et al. 2004). This effect is attributed to the inhibition of essential cellular functions, driving cells into a dormant, non-replicating state (Keren et al. 2004; Wang and Wood 2011). On the other hand, the deletion of the antitoxin PA14_51020 in *P. aeruginosa* PA14 increased persister cell counts under tobramycin stress. Further analysis showed that the toxin PA14_51010 is involved in bacterial persistence by reducing NAD + levels (Zhou et al. 2021). Another system involved in the persister cells phenotype is the stringent response, which is primarily mediated by the alarmone guanosine pentaphosphate/tetraphosphate [(p)ppGpp] (Pacios et al. 2020). ppGpp interacts with RNA polymerase and alters the expression of diverse genes (Pacios et al. 2020), and its production is mediated by RelA and SpoT in response to stress conditions (Pausch et al. 2020). In *P. aeruginosa*, *spoT* mutant strains showed elevated ppGpp levels and enhanced survival under quinolone stress (Viducic et al. 2006). Similarly, in *Bacillus subtilis*, ppGpp accumulation selected persister cells by depleting intracellular GTP, a crucial metabolite for replication and growth (Fung et al. 2025). Lastly, the SOS response, essential for DNA repair and mediated by the transcriptional repressor LexA and the inducible factor RecA, has also been associated with persister cells (Maslowska et al. 2019; Podlesek and Žgur Bertok 2020). Under stress, such as DNA damage, RecA mediates the cleavage of LexA and regulates the transcription of genes involved in, for example, DNA repair, cell arrest, and SOS response (Kovačič et al. 2013; Podlesek and Žgur Bertok 2020).

Although significant progress has been made in understanding bacterial persistence mechanisms, much less is known about how persistence and population recovery affect the virulence potential of *P. aeruginosa* and its interaction with the host. In particular, the physiological adaptations that occur after exposure to different stressors remain poorly characterized. In this study, we investigated the persister cells in *P. aeruginosa* following exposure to the bactericidal antibiotics imipenem, a β-lactam that binds to penicillin-binding proteins (PBPs), inhibiting the final stages of peptidoglycan synthesis and compromising cell wall integrity (Rodloff et al. 2006), and the fluoroquinolone ciprofloxacin, which primarily targets DNA gyrase and topoisomerase IV, enzymes essential for DNA replication and transcription, leading to lethal double-strand breaks (Drlica and Zhao 1997; Shariati et al. 2022). These drugs, which are widely used to treat *P. aeruginosa* infections (Soares et al. 2019; Alhajj et al. 2022), were selected since they represent distinct stress types, enabling us to assess whether the nature of the inducing stress influences persister cell physiology and virulence potential. We then performed a phenotypic characterization of these persister cells. By comparing persister and recovered populations in planktonic, biofilm, and intracellular states, we sought to provide new insights into the pathogenic potential of *P. aeruginosa* persisters under distinct stress conditions.

## Materials and methods

### Bacterial strains, cell lineage, and growth conditions

*Pseudomonas aeruginosa* PA14 reference strain, kindly provided by Dr. Daniel de Assis Santos (Peres-Emidio et al. 2022), and the clinical isolates 12-0048 and 16-0040, obtained from the Hospital Risoleta Tolentino Neves (HRTN), Belo Horizonte, Minas Gerais, Brazil (Igídio et al. 2025), were used in this study. Isolate 16-0040 was obtained from the tracheal aspirate and isolate 12-0048 from the bronchoalveolar lavage of patients admitted to the Intensive Care Unit of the HRTN. Bacterial strains were maintained on *Pseudomonas isolation agar* (PIA) (Neogen, USA) and grown overnight (~ 18 h) at 37 °C under shaking conditions (120 rpm) in Lysogeny broth (LB) (USB Corporation, USA). Stock solutions of imipenem (Nova Farma, Brazil), ciprofloxacin (Drogavet, Brazil), and gentamicin (Gentatec, Brazil) were prepared in distilled water and stored at 4 °C for up to two weeks. 5-Fluorouracyl (5-FU) was purchased from Libbs Farmacêutica.

Phagocytosis experiments were performed using AMJ2-c11 alveolar macrophages, derived from mouse lungs, and obtained from the Rio de Janeiro Cell Bank (Brazil). Cells were thawed from frozen aliquots stored at -80 °C and cultured in DMEM (Dulbecco’s Modified Eagle’s Medium) high-glucose (Cultilab, Brazil) supplemented with 10% fetal bovine serum (FBS) at 37 °C in a 5% CO₂ atmosphere.

### Minimal inhibitory concentration (MIC)

The minimum inhibitory concentration (MIC) was determined by the broth microdilution method in Mueller–Hinton (MH) broth, as previously described (Wiegand et al. 2008). Briefly, serial two-fold dilutions of imipenem (0.125–32 μg/mL), ciprofloxacin (0.031–16 μg/mL), gentamicin (0.031–16 μg/mL), and hydrogen peroxide (Synth, USA; H_2_O_2_) (0.0098–2.5%) were prepared in MH broth and inoculated with *P. aeruginosa* (final cell concentration of ~ 1 × 10^6^ CFU/mL). Plates were incubated for 24 h at 37 °C, and the MIC was defined as the lowest concentration of antimicrobial compound that visually inhibited bacterial growth.

### Determination of the antibiotic concentration associated with persister cells

To determine antibiotic concentrations at which persister cells can be detected, stationary-phase *P. aeruginosa* PA14 was grown overnight (~ 18 h) in LB, and the OD was adjusted to 0.1 (~ 1 × 10^8^ CFU/mL). Cells were then exposed to imipenem or ciprofloxacin at 1, 3, 10, 30, and 100 × MIC for 4 h. Cells were collected by centrifugation, washed twice with saline to remove residual antibiotic, serially diluted, plated out on LB agar plates, and incubated for 24 h at 37 °C. Untreated cells were used as controls.

### Persister cells assay

**Fig. 1:**
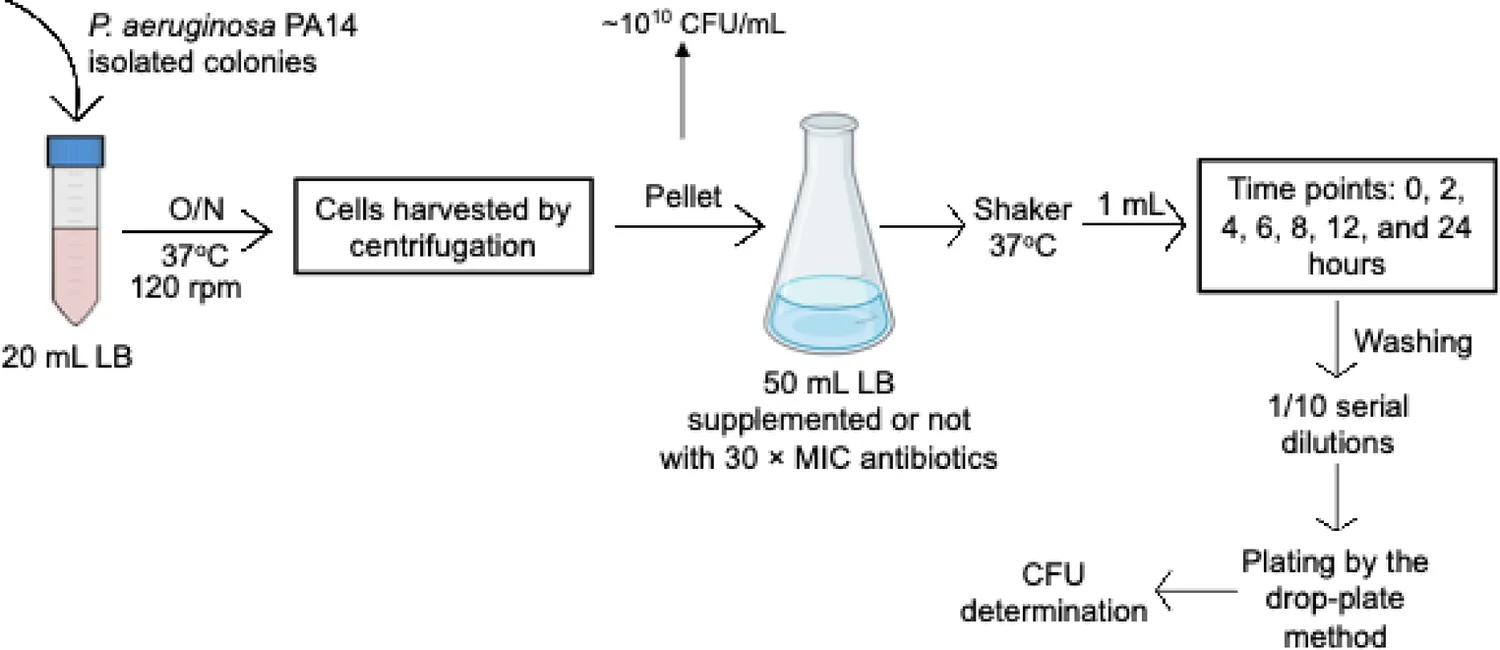
Schematic representation of the persister cell assay workflow. *P. aeruginosa* PA14 was grown overnight in LB medium to the stationary phase, harvested by centrifugation, and the complete bacterial pellet was resuspended in fresh LB. The resulting concentrated suspension was incubated with or without antibiotics (30 × MIC imipenem (60 µg/mL) or ciprofloxacin (3.75 µg/mL)). At the indicated time points, 1 mL samples were collected, washed, serially diluted, and plated for CFU enumeration

Initially, the MIC for PA14 was determined to be 2 µg/mL for imipenem and 0.125 µg/mL for ciprofloxacin. Then, stationary-phase overnight (~ 18 h) cultures of *P. aeruginosa* PA14 grown in LB at 37 °C with shaking were centrifuged, the bacterial pellet was resuspended in 50 mL of LB containing 30 × MIC imipenem (60 µg/mL) or ciprofloxacin (3.75 µg/mL), and transferred to Erlenmeyer flasks, which were incubated at 37 °C for 24 h under shaking conditions (120 rpm). For biphasic killing analysis, 1 mL samples were collected at 0, 2, 4, 6, 8, 12, and 24 h, washed to remove residual antibiotic, serially diluted in 0.9% saline, and plated out on LB agar. Untreated cells were grown as controls (Fig. 1).

To obtain recovered populations, PA14 grown overnight (~ 18 h) was collected by centrifugation, resuspended in LB containing 30 × MIC of imipenem (60 µg/mL) or ciprofloxacin (3.75 µg/mL), and incubated at 37 °C under shaking conditions (120 rpm) for 24 h. The cells were then harvested, washed with saline solution to remove residual antibiotic, and transferred to LB medium for 24 h at 37 °C and shaking.

### Persistence by the Replica Plating Tolerance Isolation System (REPTIS) model

The REPTIS method was conducted as previously described (Matsuo et al. 2019), with minor modifications. Overnight (~ 18 h) stationary-phase *P. aeruginosa* PA14 cultures grown in LB at 37 °C and 120 rpm were adjusted to an OD_600nm_ of 0.1 (~ 1 × 10^8^ CFU/mL), and 100 µL was spread onto LB agar containing 30 × MIC imipenem (60 µg/mL) or ciprofloxacin (3.75 µg/mL). The plates (“master plates”) were incubated at 37 °C for 72 h with daily monitoring. Then, the content from master plates was collected using a saline-soaked swab and transferred to antibiotic-free LB agar (“replica plates”), which were incubated for 24 h at 37 °C, to allow the growth of surviving cells. The swab contents were also serially diluted and plated to quantify the number of cells that recovered from antibiotic exposure. Indicative of persistence was the absence of growth on the master plates in the presence of antibiotics, followed by growth on the replica plates after transfer to antibiotic-free medium.

### Revival assay

Recovery of persister cells was evaluated by growth curves in LB. Overnight (~ 18 h) stationary-phase *P. aeruginosa* PA14 grown in LB at 37 °C with shaking were centrifuged, and the bacterial pellet was resuspended in LB containing 30 × MIC of imipenem (60 µg/mL) or ciprofloxacin (3.75 µg/mL). The cells were incubated for 24 h at 37 °C with shaking (120 rpm). Subsequently, the cultures were washed to remove residual antibiotic, serially diluted in 0.9% saline, and resuspended in LB. Then, 100 µL of bacterial inoculum was dispensed into 96-well plates and incubated at 37 °C for 20 h, with OD_600nm_ measured hourly in a microplate reader.

### Cell viability by flow cytometry

Cell viability of imipenem- and ciprofloxacin-persistent PA14 cells was assessed by flow cytometry using the BacLight™ RedoxSensor™ Green Vitality Kit (Thermo Fisher). Overnight (~ 18 h) stationary-phase *P. aeruginosa* PA14 grown in LB at 37 °C with shaking were centrifuged, and the bacterial pellet was resuspended in LB containing 30 × MIC of imipenem (60 µg/mL) or ciprofloxacin (3.75 µg/mL) at 37 °C with shaking (120 rpm). Following antibiotic exposure, cells were washed twice, resuspended in filtered PBS to an OD_600nm_ of 0.1 (~ 1 × 10^8^ CFU/mL), and stained with 1 µL RedoxSensor™ Green (RSG) and 1 µL propidium iodide (PI) for 10 min at 37 °C in the dark. Cells were fixed with 2% formaldehyde for 20 min and analyzed on a BD FACSCanto II, with between 1,000 and 50,000 events recorded per sample, depending on cell recovery. Samples were collected after 2, 8, and 24 h of antibiotic exposure.

Furthermore, 24-h recovered cells were analyzed. For this, PA14 grown overnight (~ 18 h) was collected by centrifugation, resuspended in LB containing 30 × MIC imipenem (60 µg/mL) or ciprofloxacin (3.75 µg/mL), and incubated at 37 °C under shaking conditions (120 rpm) for 24 h. The cells were then harvested, washed with saline solution to remove residual antibiotic, and transferred to LB medium for 24 h at 37 °C and shaking. The percentage of cells was normalized based on the gated percentage and the initial inoculum (~ 10^8^ CFU/mL).

Ethanol-killed cells stained with PI were used as dead controls, and unstained and RSG-stained cells served as live controls.

### Gene expression by qRT-PCR

*P. aeruginosa* PA14 was grown overnight (~ 18 h) in 50 mL LB until the stationary phase, then the cells were collected by centrifugation and exposed to 30 × MIC imipenem (60 µg/mL) or ciprofloxacin (3.75 µg/mL) (final volume of 50 mL) for 8 h. Then, the cells were washed to remove residual antibiotic (persister cells), and the RNA was extracted, or the cells were cultivated in LB for 24 h to obtain recovered cells. Total RNA from persistent and recovered PA14 cells was extracted using the RNeasy Mini Kit (Qiagen) and quantified by NanoDrop. cDNA was synthesized using the iScript™ cDNA Synthesis Kit (Bio-Rad). Gene expression of *recA, relA, higB, pvdS, pqsA, spoT,* and *lon* was assessed by qRT-PCR, with 16S rRNA used as the reference gene. Relative expression was calculated using the 2^−ΔΔCt^ method. Primer sequences are available upon request.

### Persistence by the static biofilm model

Persistence in planktonic and adherent cells was assessed using a static biofilm model as described by (Liao et al. 2024), with minor modifications. Overnight (~ 18 h) cultures of *P. aeruginosa* PA14 and clinical isolates 12-0048 and 16-0040 grown in LB, 37 °C, and 120 rpm were adjusted to an OD_600nm_ of 0.1 in LB, and 1 mL of the bacterial inoculum was transferred to 12-well plates. Plates were incubated statically at 37 °C for 24 h. The media containing non-adherent planktonic cells were collected and transferred to microtubes. Biofilms were washed twice with 0.9% saline, detached by vigorous pipetting, and the cells were transferred to microtubes. Both populations (i.e., planktonic and biofilm cells) were washed, resuspended in 1 mL of LB containing 30 × MIC imipenem (60 µg/mL) or ciprofloxacin (3.75 µg/mL), and incubated for 4 h at 37 °C. Cells were washed, serially diluted in saline, and plated out on LB agar using the drop-plate method (Herigstad et al. 2001), in which three drops of each dilution are placed on agar plates to determine CFU. Plates were incubated for 24 h at 37 °C for CFU enumeration. Untreated cultures served as controls.

### Pigment production

Pyoverdine, pyocyanin, and pyorubin production were quantified spectrophotometrically, as previously described (Lo et al. 2016; Ferreira et al. 2018) in persistent and recovered *P. aeruginosa* PA14 prepared as described in Section "Persister cells assay". Twenty-four-hour PA14 cultures were centrifuged (5,000 × g for 10 min), and supernatants were collected. Pyoverdine was measured at 404 nm. Pyocyanin and pyorubin were quantified by chloroform extraction. Five mL of culture supernatant was mixed with 3 mL chloroform, vortexed, and centrifuged (1,000 × g for 5 min). The absorbance of the aqueous phase (upper) was measured at 520 nm for pyorubin. For pyocyanin, 1 mL of 0.2 M HCl was added to the organic phase (blue), and the absorbance of the resulting pink phase was measured at 520 nm.

### Biofilm biomass quantification

Biofilm biomass was quantified using the crystal violet method as previously described (O’Toole et al. 1999). For persister cells, PA14 stationary-phase cells were grown overnight (~ 18 h) in 20 mL LB at 37 °C and 120 rpm. Cells were collected by centrifugation, resuspended in 50 mL of LB containing 30 × MIC concentrations of imipenem (60 µg/mL) or ciprofloxacin (3.75 µg/mL), transferred to Erlenmeyer flasks, and incubated for 24 h at 37 °C. For recovered cells, antibiotic-exposed cells were washed twice to remove residual antibiotic, resuspended in LB, and incubated for 24 h at 37 °C. Persistent and recovered cultures were adjusted to an OD_600nm_ of 0.1, and 100 µL was transferred to flat-bottom 96-well plates, which were incubated statically at 37 °C for 24 h. Wells were washed with water, stained with 0.1% crystal violet for 10 min, washed, and then treated with 99% ethanol for 20 min. Absorbance was recorded at 595 nm.

### Congo red assay

Extracellular matrix production was assessed using the Congo Red binding assay. For persister cells, PA14 stationary-phase cells were grown overnight (~ 18 h) in 20 mL LB at 37 °C and 120 rpm. Cells were collected by centrifugation, resuspended in 50 mL of LB containing 30 × MIC concentrations of imipenem (60 µg/mL) or ciprofloxacin (3.75 µg/mL), transferred to Erlenmeyer flasks, and incubated for 24 h at 37 °C. For recovered cells, persister cells were washed twice to remove residual antibiotic, resuspended in LB, and incubated for 24 h at 37 °C. Persistent and recovered cells grown for 24 h were washed, and the OD_600nm_ was adjusted to 0.1. Then, 10 µL of the inoculum was spotted onto LB agar plates supplemented with 40 mg/L Congo Red (Neon, Brazil). The plates were incubated at 37 °C for 24 h and photographed. Images were analyzed using ImageJ. First, they underwent automatic balance adjustment; then, an oval region of interest (ROI) was defined for each colony, and the mean gray value was measured across all samples. Identical ROI size and measurement parameters were used for all images. The mean values were recorded and used to create the graph on GraphPad Prism.

### Hydrogen peroxide sensitivity assay

To evaluate the effect of H_2_O_2_ on persistence, the H_2_O_2_ sensitivity assay was performed. Overnight (~ 18 h) stationary-phase *P. aeruginosa* PA14 cultures grown in LB at 37 °C and 120 rpm were adjusted to an OD_600nm_ of 0.1 and exposed to 0.4% H_2_O_2_ for 1 h. Cells were washed and treated with 30 × MIC of imipenem (60 µg/mL) or ciprofloxacin (3.75 µg/mL) at 37 °C for 24 h with shaking. After treatment, cultures were washed, resuspended in saline, serially diluted, and plated out on LB agar for CFU enumeration. Cultures treated with antibiotics without prior H_2_O_2_ exposure served as controls.

### Effect of DNA damage on persistence acquisition

To evaluate the effect of DNA damage on persistence, cells were pretreated with 5-fluorouracil (5-FU). Overnight (~ 18 h) stationary-phase *P. aeruginosa* PA14 cultures grown in LB at 37 °C were exposed to sub-MIC of 5-FU (256 µg/mL) for 4 h, washed twice with 0.9% saline, and resuspended in LB containing 30 × MIC imipenem (60 µg/mL) or ciprofloxacin (3.75 µg/mL). Cultures were incubated at 37 °C for 24 h with shaking (120 rpm), washed, serially diluted, and plated out on LB agar for CFU enumeration.

### Persister cell detection post-phagocytosis

Persistence following phagocytosis was assessed using AMJ2-c11 alveolar macrophages. Cells were cultured in DMEM high-glucose medium containing 10% FBS at 37 °C and 5% CO_2_ until ~ 80% confluence. Non-adherent and adherent cells were collected, harvested, adjusted to 3 × 10^6^ cells/well in DMEM, and seeded (2 mL/well) into 6-well plates for 24 h at 37 °C in a 5% CO₂ atmosphere. *P. aeruginosa* PA14 overnight (~ 18 h) stationary-phase cultures were washed, adjusted to 3 × 10^7^ CFU/mL (MOI = 10; 3 × 10^7^ CFU/mL bacteria/3 × 10^6^ macrophages), and added to macrophages for 30 min to allow phagocytosis. Gentamicin (100 µg/mL) was added for 30 min to eliminate extracellular bacteria. Macrophages were lysed with 0.1% Triton X-100, and 100 μL of the resulting solution was collected. Serial dilutions were prepared and plated out on LB agar plates, which were then incubated at 37 °C for 24 h. This step was used to determine the number of cells recovered from phagocytosis and to establish the initial cell concentration for the subsequent persistence assay.

Cells recovered after phagocytosis were then exposed to 30 × MIC imipenem (60 µg/mL) or ciprofloxacin (3.75 µg/mL) and incubated at 37 °C, 120 rpm for 24 h. Samples were centrifuged, washed twice, serially diluted in saline, and plated out on LB agar. The percentage survival of cells after antibiotic exposure was calculated based on the cell numbers determined after phagocytosis, as mentioned above.

### Statistical analysis

Statistical analyses were performed using GraphPad Prism (version 10.0, San Diego, USA). All experiments were conducted in at least three independent biological replicates, and the results were expressed as the mean ± standard deviation (SD). The Shapiro–Wilk test was applied to assess the normality of the data. Parametric data was analyzed by One-way ANOVA with Tukey’s or Dunnett’s post-hoc tests for multiple comparisons, and Student’s t-test for pairwise comparisons. For non-parametric data, comparisons between two groups were performed using either the t-test or the Mann–Whitney test. Results were considered statistically significant when p < 0.05.

## Results

### Antimicrobial susceptibility of *P. aeruginosa*

The antimicrobial susceptibility of *P. aeruginosa* PA14 and the clinical isolates 12-0048 and 16-0040 were determined by the MIC assay in MH broth (Table 1). According to the Clinical and Laboratory Standards Institute (CLSI) (CLSI 2020), all *P. aeruginosa* strains were either susceptible or showed intermediate susceptibility to imipenem, with MICs ranging from 2 μg/mL for PA14 WT and the isolate 16-0040 to 4 μg/mL for the isolate 12-0048. Similarly, all strains were either susceptible (PA14 and 12-0048) or intermediately susceptible (16-0040) to ciprofloxacin, with MICs ranging from 0.125 μg/mL (PA14) to 1 μg/mL (16-0040).

**Table 1:** Minimal inhibitory concentration of *P. aeruginosa* PA14 WT and clinical isolates

Bacterial strain	MIC (μg/mL)	
	Imipenem	Ciprofloxacin
PA14 WT	2	0.125
12-0048	4	0.25
16-0040	2	1 (R)

Experiments were performed in at least three biological replicates. (R): resistant

### Persister cells are detected at 30 × MIC of imipenem and ciprofloxacin

**Fig. 2:**
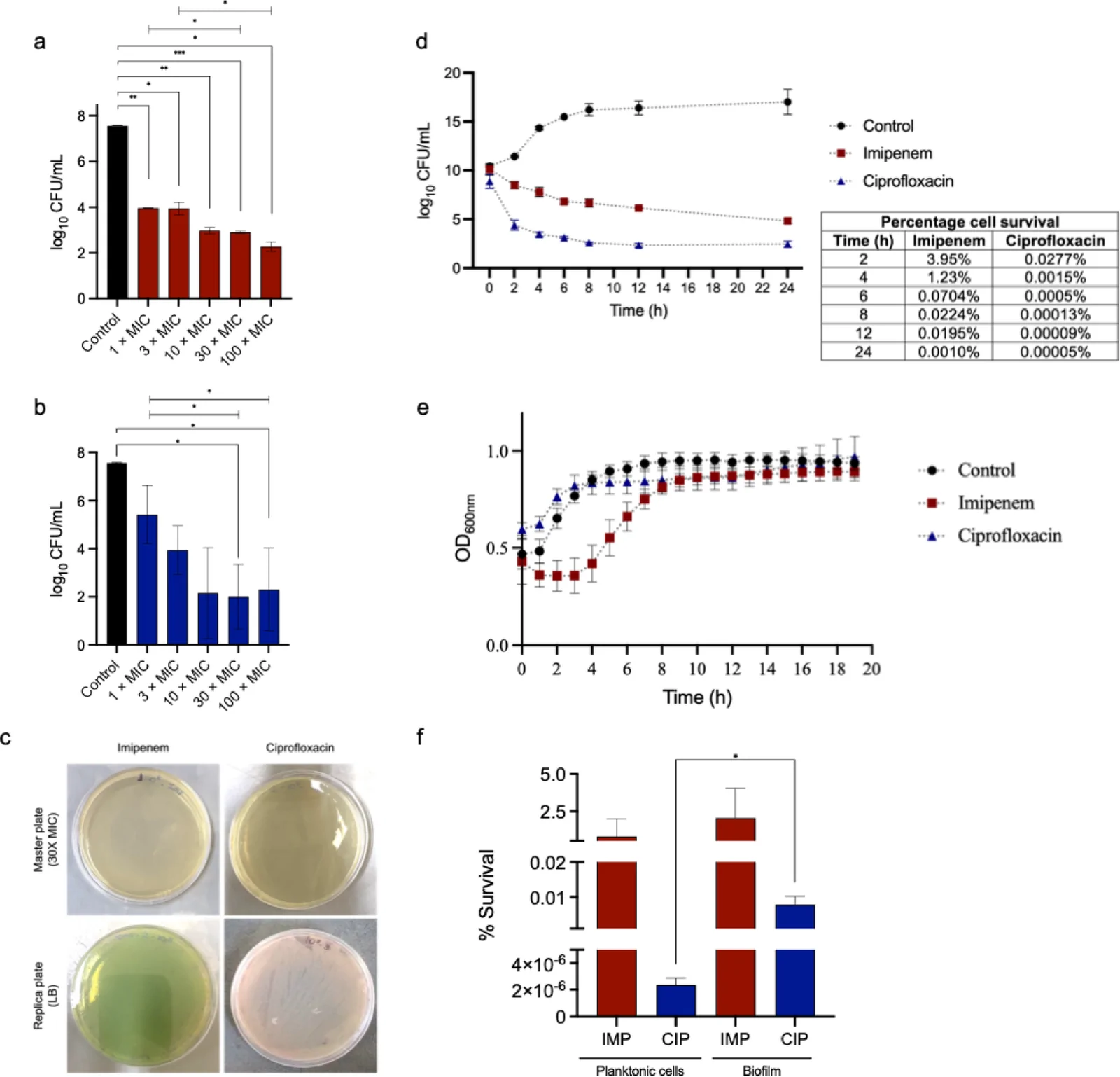
*P. aeruginosa* PA14 persister cells are detected after imipenem and ciprofloxacin treatment. Evaluation of **a** imipenem and **b** ciprofloxacin concentrations at which persister cells are detected. *P. aeruginosa* PA14 at 0.1 OD_600nm,_ grown overnight (~ 18 h), was treated with 1, 3, 10, 30, and 100 × MIC of imipenem or ciprofloxacin for 4 h, and CFU/mL was determined. **c** Persister cell detected by the REPTIS method. The OD_600nm_ of PA14 grown overnight (~ 18 h) in LB was adjusted to 0.1 (~ 10^8^ CFU/mL), and cells were plated out on LB agar plates containing 30 × MIC of imipenem (60 µg/mL) or ciprofloxacin (3.75 µg/mL) for 72 h (master plates). The contents of the master plates were then transferred to antibiotic-free LB agar plates (replica plates) for 24 h. **d** Biphasic curve of *P. aeruginosa* persister cells. PA14 cultures grown overnight (~ 18 h) were treated with 30 × MIC of imipenem or ciprofloxacin (60 or 3.75 μg/mL, respectively) for 0, 2, 4, 6, 8, 12, and 24 h, and the CFU/mL was determined. **e** Revival assay. PA14 persister cells were washed and grown in LB for 24 h at 37 °C. The OD_600nm_ was measured every hour for 20 h in a microplate reader. **f** Persister cells in non-attached and attached populations upon imipenem and ciprofloxacin exposure. Biofilms of *P. aeruginosa* were grown on 12-well plates for 24 h in LB at 37 °C and static conditions. Non-attached (planktonic) cells were collected and transferred to microtubes, and biofilms (attached cells) were washed with saline solution, and the cells were collected by vigorous pipetting. Both cell populations were treated with 30 × MIC of imipenem (60 µg/mL) or ciprofloxacin (3.75 µg/mL) for 4 h, and the CFU/mL was determined. IMP: imipenem; CIP: ciprofloxacin. All experiments were conducted in at least three independent biological replicates. The Shapiro–Wilk test was applied to assess data normality, and the Student’s t-test for pairwise comparisons. *p < 0.05; **p < 0.01; ***p < 0.001; ****p < 0.0001

Persistence is characterized by a small subpopulation of cells that withstands high antibiotic pressure at concentrations well above the MIC (Balaban et al. 2019; Ovsepian et al. 2020; Patel et al. 2022). To determine the concentrations of imipenem or ciprofloxacin at which persister cells are detected, *P. aeruginosa* PA14 cultures grown overnight (~ 18 h) were exposed to 1, 3, 10, 30, and 100 × MIC for 4 h. As shown in Fig. 2a, treatment with 10, 30, or 100 × MIC did not result in significant changes in cell counts. For imipenem, log_10_ CFU/mL values were 2.98, 2.9, and 2.3 at 10, 30, and 100 × MIC, respectively, compared to 7.6 in the untreated control, with survival of 0.025, 0.027, 0.0027, 0.0022, and 0.0005%, respectively (Fig. 2a). Ciprofloxacin reduced cell counts from log_10_ CFU/mL 7.6 to 5.4, 3.9, 2.1, 2.0, and 2.3 at 1, 3, 10, 30, and 100 × MIC, respectively. This represents survival percentages of 3.7, 0.05, 0.004, 0.001, and 0.002% at 1, 3, 10, 30, and 100 × MIC, respectively (Fig. 2b). Based on these results, all subsequent persistence experiments were performed using 30 × MIC of imipenem and ciprofloxacin.

First, we detected *P. aeruginosa* PA14 persister cells after exposure to 30 × MIC of imipenem and ciprofloxacin by the REPTIS method, adapted from Matsuo et al. (2019). *P. aeruginosa* PA14 at 10^8^ CFU/mL was cultured for 72 h on LB agar plates containing 30 × MIC of imipenem (60 µg/mL) or ciprofloxacin (3.75 µg/mL) (master plates). At this stage, no bacterial growth is expected (Matsuo et al. 2019), except for resistant mutants. The content of the master plates was then transferred using a sterile swab to LB agar plates without antibiotics (replica plates), which were incubated for 24 h. Because persister cells can resume growth once antibiotic stress is removed, colonies growing on the replica plates were considered indicative of persistence. As shown in Fig. 2c, no growth was observed on the master plates for either antibiotic treatment, whereas extensive growth was observed on the corresponding replica plates. Interestingly, while colonies that recovered from imipenem exposure exhibited the typical green pigmentation of *P. aeruginosa*, those that survived ciprofloxacin treatment showed a brownish pigmentation (Fig. 2c), potentially indicating differences in pigment production. After growth on replica plates, ciprofloxacin-exposed PA14 showed a percentage survival of 0.02%, and imipenem-treated cells of 0.018% (Supplementary Figure S1).

To further support our observation that persister cells can be detected at 30 × MIC of imipenem and ciprofloxacin, we next evaluated the killing dynamics of *P. aeruginosa* PA14 exposed to these antibiotics. Stationary-phase planktonic cells grown overnight were treated with 30 × MIC of imipenem or ciprofloxacin (60 or 3.75 μg/mL, respectively) for 0, 2, 4, 6, 8, 12, and 24 h, and the CFU/mL was determined. In both antibiotic-treated cells, a biphasic killing curve was observed, characteristic of persister cell detection (Balaban et al. 2019) (Fig. 2d). Treatment with imipenem led to a rapid decrease in viable cells during the first 4 h, with a survival percentage of 1.23% compared to the initial cell concentration. After this initial killing phase, the bacterial population showed lower reduction, remaining relatively stable from 6 to 24 h with a reduction of approximately 2 logs (log_10_ CFU/mL of 6.8 at 6 h and 4.8 at 24 h). After 24 h, 0.001% of the bacterial population survived imipenem treatment. On the other hand, exposure to ciprofloxacin resulted in a sharper initial reduction, with log_10_ CFU/mL decreasing from approximately 8.9 to 4.4 within the first two hours (4.5 logs reduction; 0.0277% survival), followed by a reduction to 3.4 at 4 h post-treatment (0.0015% survival). As observed with imipenem, this rapid killing phase was followed by a plateau, indicating that a small fraction of the population survived despite prolonged antibiotic exposure. After 24 h, 0.00005% of the cells survived ciprofloxacin treatment (Fig. 2d).

Together, the CFU analyses at different lethal concentrations, the REPTIS assay on solid media, and the biphasic killing patterns in liquid cultures confirm the detection of persister cells of *P. aeruginosa* PA14 following exposure to 30 × MIC of imipenem or ciprofloxacin.

### Revival and antibiotic susceptibility confirm the persister phenotype

A key characteristic of persister cells is their ability to resume growth once the stress is removed (Balaban et al. 2019). Furthermore, it is also essential to distinguish between persistence and heritable resistance when analyzing surviving populations. To address this, we performed a revival assay by monitoring the growth curves of *P. aeruginosa* PA14 persister cells in LB over 20 h (Fig. 2e) by OD_600nm_ measurement. This allowed us to evaluate the duration of the lag phase and the time required for cells surviving imipenem or ciprofloxacin exposure to recover and resume growth. Imipenem-persistent cells exhibited a 4-h lag phase, whereas ciprofloxacin-persistent cells showed a shorter growth delay, similar to the control (~ 1 h) (Fig. 2e).

Then, to confirm that the cells are persisters rather than resistant strains, the MIC was determined by the broth microdilution method in MH broth for imipenem, ciprofloxacin, and gentamicin. Furthermore, the MIC of the recovered cells (i.e., persister cells that were washed to remove residual antibiotics and allowed to resume growth in LB for 24 h) was determined to evaluate whether antibiotic exposure had selected for heritable resistance (Table 2). As persister cells are defined as transient phenotypic variants, they are expected to retain the same antimicrobial susceptibility profile as the parental strain once the stress is removed. For susceptibility analyses, only changes greater than twofold in MIC values are considered significant (Mouton et al. 2018b, a; Kadeřábková et al. 2024). Overall, the persister and recovered populations exhibited MICs similar to those of the parental strain for the tested antimicrobial agents (Table 2). Our findings reinforce that the observed tolerant phenotype is due to persistence rather than acquired resistance.

**Table 2:** Minimal inhibitory concentration of imipenem and ciprofloxacin for *P. aeruginosa* PA14 persistent and recovered cells

Bacteria	Antimicrobial agent		
	Imipenem (μg/mL)	Ciprofloxacin (μg/mL)	Gentamicin (μg/mL)
Control	2	0.125	2
Imipenem-persistent cells	2	0.125	1
Imipenem recovered cells	4	0.25	2
Ciprofloxacin-persistent cells	2	0.125	1
Ciprofloxacin recovered cells	4	0.125	2

Experiments were performed in at least three biological replicates

### Persistence in adhered and non-adhered cells

Given that surface-attached bacteria are phenotypically distinct from planktonic cells (Hernández-Jiménez et al. 2013), account for the majority of *P. aeruginosa* infections (Tuon et al. 2022), and harbor persistent cells that contribute to antibiotic recalcitrance (Zadeh et al. 2022), we used the static biofilm model to assess persistence in both biofilm-attached and planktonic (non-attached) cells upon exposure to imipenem and ciprofloxacin (Liao et al. 2024). Biofilms of *P. aeruginosa* were grown on 12-well plates for 24 h in LB, after which non-adhered and adhered cells were collected, and treated with 30 × MIC of imipenem (60 µg/mL) or ciprofloxacin (3.75 µg/mL) for 4 h, and the CFU/mL was determined. In addition to *P. aeruginosa* PA14 (Fig. 2f), a well-characterized biofilm-forming strain, clinical isolates 12-0048 and 16-0040 were included to assess strain-specific variability and enhance the clinical relevance (Supplementary Figure S2).

Figure 2f shows the percentage survival of planktonic and attached (biofilm) cells after treatment with imipenem and ciprofloxacin, calculated as the ratio between the cell numbers in the treated and untreated control conditions. Overall, persister cells were detected in both planktonic and biofilm populations, with a higher incidence observed in biofilms treated with ciprofloxacin (Fig. 2f). Survival percentages of 0.8% and 2.04% were obtained for planktonic and biofilm cells treated with imipenem, respectively. In contrast, ciprofloxacin presented a more pronounced effect, with survival percentages of 2.4 × 10^–6^ and 0.007% for planktonic and biofilm cells, respectively.

Persister cell detection differed between attached and non-attached cells in clinical isolates. Isolate 12-0048 (Supplementary Figure S2a) was exposed to imipenem and ciprofloxacin, while isolate 16-0040 (Supplementary Figure S2b) was tested only with imipenem due to its ciprofloxacin resistance (Table 1). Isolate 12-0048 exhibited higher persister levels in biofilms exposed to ciprofloxacin, consistent with the PA14 strain, while imipenem-treated 16-0040 presented high persister levels in biofilms. Overall, these results confirm the presence of persisters upon imipenem and ciprofloxacin in both adhered and non-adhered cells.

### Cell viability of persister cells by flow cytometry

**Fig. 3:**
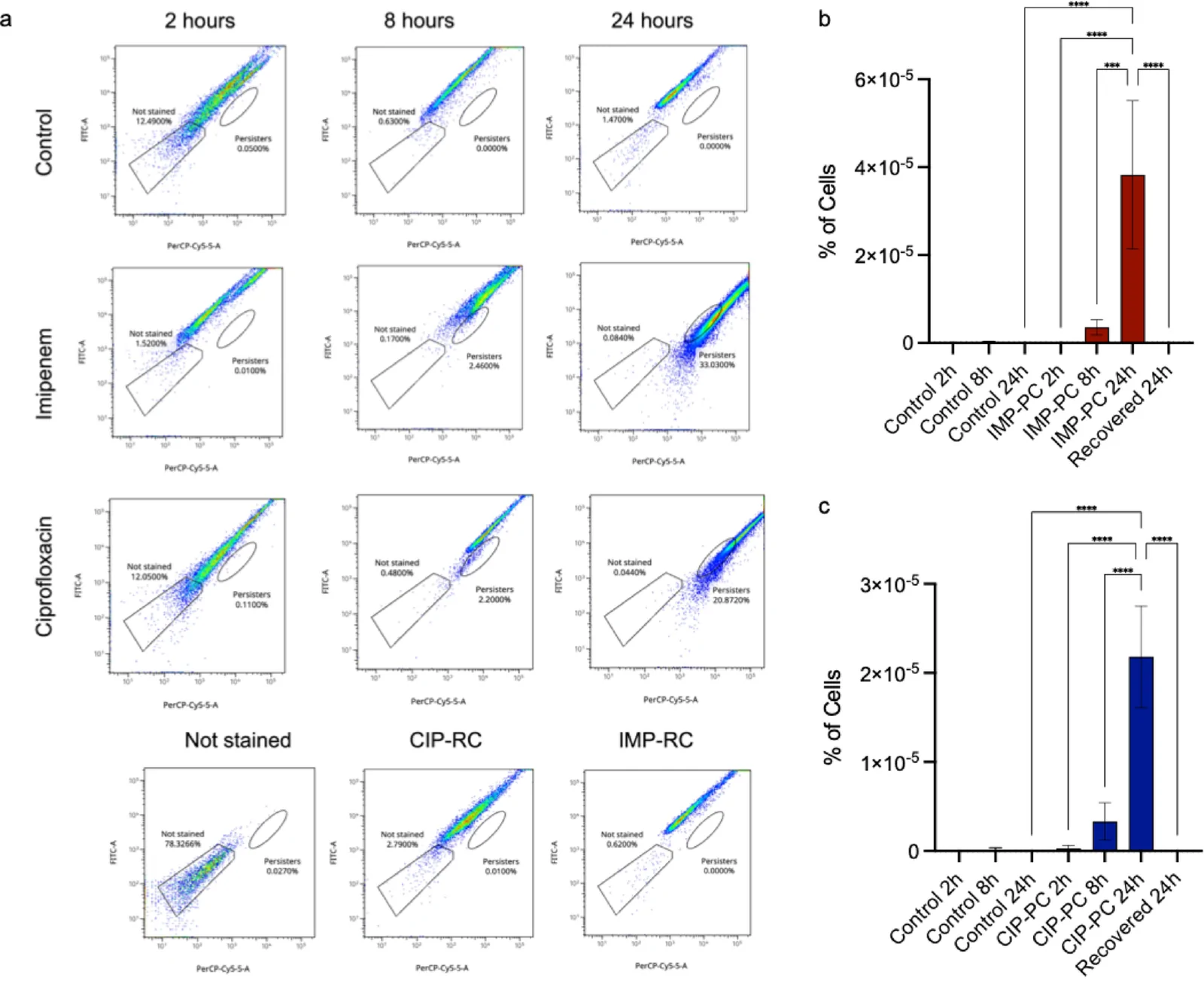
Cell viability of persister cells assessed by flow cytometry. **a** Flow cytometry plots. Percentage of persister PA14 cells in **b** imipenem and **c** ciprofloxacin-treated populations. *P. aeruginosa* grown overnight (~ 18 h) in LB was cultivated in LB supplemented with 30 × MIC of imipenem (60 μg/mL) or ciprofloxacin (3.75 μg/mL) for 2, 8, and 24 h at 37 °C. Cells were washed, diluted to an OD_600nm_ of 0.1 (~ 10^8^ CFU/mL), and stained with 1 μL of RSG and 1 μL of PI for 10 min at 37 °C. Flow cytometry was performed using a BD FACSCanto II cytometer. Recovered cells grown for 24 h were also analyzed. The percentage of cells was normalized based on the gated percentage and the initial inoculum (~ 10^8^ CFU/mL). *IMP-PC* imipenem-persistent cells, *CIP-PC* ciprofloxacin-persistent cells, *CIP-RC* cells that recovered from ciprofloxacin treatment, *IMP-RC* cells that recovered from imipenem treatment. All experiments were conducted in at least three independent biological replicates. The Shapiro–Wilk test was applied to assess data normality, followed by One-Way ANOVA. ***p < 0.001; ****p < 0.0001

Cell viability and metabolic state of persister cells were assessed by flow cytometry using the BacLight™ RedoxSensor™ Green Vitality Kit, which differentiates cells based on redox activity (RSG, FITC channel) and membrane integrity (PI, PerCP-Cy5.5 channel). Persister cells were defined as exhibiting low fluorescence for both RSG and PI. In the flow cytometry plots (Fig. 3a), this population was localized just below the main RSG⁺/PI⁻ (viable) cluster and was conservatively gated to exclude highly PI-positive (dead) cells, metabolically active cells, and background events. The percentage survival of antibiotic-treated cells was normalized to the initial inoculum (~ 10^8^ CFU/mL) based on the percentages obtained from flow cytometry gating. The proportion of cells falling within the persister gate increased markedly over time for both antibiotics. In the control (Fig. 3a, top row), no persister cells were detected at 2, 8, or 24 h. In contrast, exposure to imipenem (Fig. 3a, second row and Fig. 3b) increased persister percentage from 10^–8^% at 2 h to 3.6 × 10^–6^% at 8 h, reaching 3.82 × 10^–4^% at 24 h (Fig. 3b). A similar pattern was observed with ciprofloxacin (Fig. 3a, third row), with an increase from 2.8 × 10^–7^% at 2 h to 3.3 × 10^–6^% at 8 h and 2.18 × 10^–5^% at 24 h (Fig. 3c). These results are consistent with our previous analysis (Fig. 3d). At 2 h, during the rapid killing phase, no persister cells were detected by flow cytometry. However, after this phase, a persistent subpopulation became detectable, coinciding with the killing plateau observed after prolonged antibiotic exposure.

### Gene expression analysis in persistent and recovered cells

**Fig. 4:**
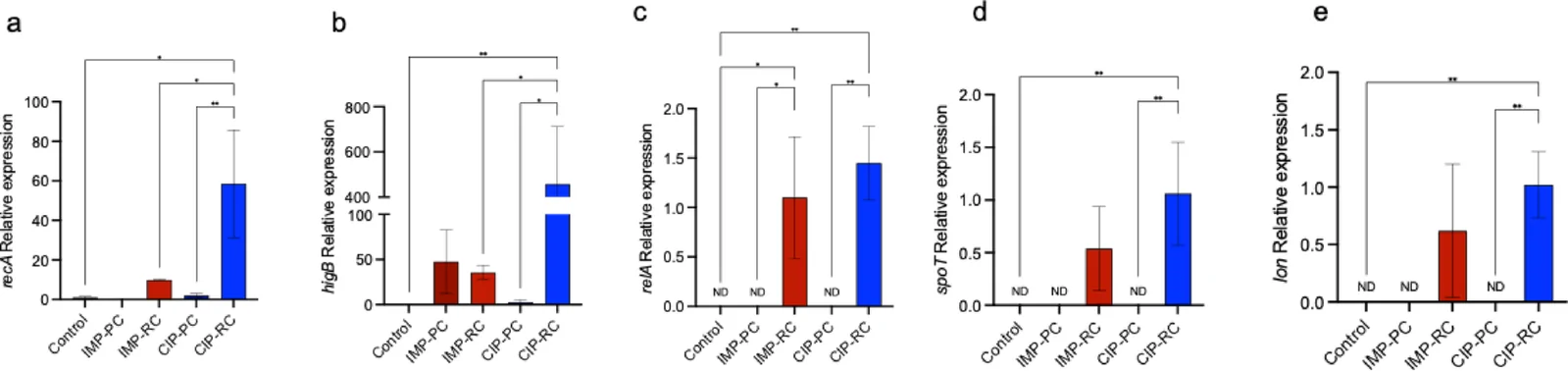
Gene expression analysis of **a** *recA*, **b** *higB*, **c** *relA*, **d** *spoT*, and **e** *lon* by qRT-PCR. PA14 persister cells were grown for 8 h in LB supplemented with 30 × MIC of imipenem (60 µg/mL) or ciprofloxacin (3.75 µg/mL), and recovered cells were grown in LB for 24 h. Control cells were also grown for 8 h. *IMP-PC* imipenem-persistent cells, *CIP-PC* ciprofloxacin-persistent cells, *CIP-RC* cells that recovered from ciprofloxacin treatment, *IMP-RC* cells that recovered from imipenem treatment, *ND* gene expression not detected after 8 h of antibiotic exposure. All experiments were conducted in at least three independent biological replicates. The Shapiro–Wilk test was applied to assess data normality, followed by a Student's t-test to compare two groups. *p < 0.05; **p < 0.01

To further investigate the mechanisms involved in persistence in *P. aeruginosa* PA14 following an 8-h exposure to imipenem and ciprofloxacin, we analyzed the expression of genes involved in SOS response (*recA*) (Fig. 4a), toxin-antitoxin system (*higB*) (Fig. 4b), and stringent response (*relA*, *spoT*, and *lon*) (Fig. 4c-e). Persistent cells were collected at 8 h as this time-point corresponds to the early stages of persister detection, as indicated by our biphasic killing curve (Fig. 2d), to capture the initial transcriptional changes associated with entry into the persister state. Furthermore, recovered cells were also included to evaluate whether gene expression returned to baseline upon resumption of growth, allowing us to distinguish transient stress responses from changes associated with persistence. For imipenem, relative *recA* expression values of 0.19 and 9.9 were obtained for persister and recovered cells, respectively. In contrast, for ciprofloxacin, values of 2.1 and 58 were observed for persister and recovered cells, respectively, indicating that *recA* induction primarily occurs upon recovery and is markedly stronger following ciprofloxacin exposure compared to imipenem (Fig. 4a). For the toxin gene *higB*, imipenem-persistent and recovered cells showed relative expression levels of 48 and 35, respectively. In ciprofloxacin-persistent cells, *higB* expression was modestly increased (2.8-fold), whereas a strong induction was observed in ciprofloxacin-recovered cells, with a relative expression of 457 (Fig. 4b). For the stringent response genes *relA, spoT*, and *lon*, no amplification was detected in the control group or in persister cells exposed to imipenem or ciprofloxacin under our experimental conditions (i.e., 8 h of antibiotic treatment). In contrast, recovered cells from these treatments showed increased expression of these genes, with ciprofloxacin-recovered cells exhibiting higher expression than imipenem-recovered cells (Fig. 4c-e).

### Exposure to stress conditions enhances antibiotic persistence in *P. aeruginosa* PA14

**Fig. 5:**
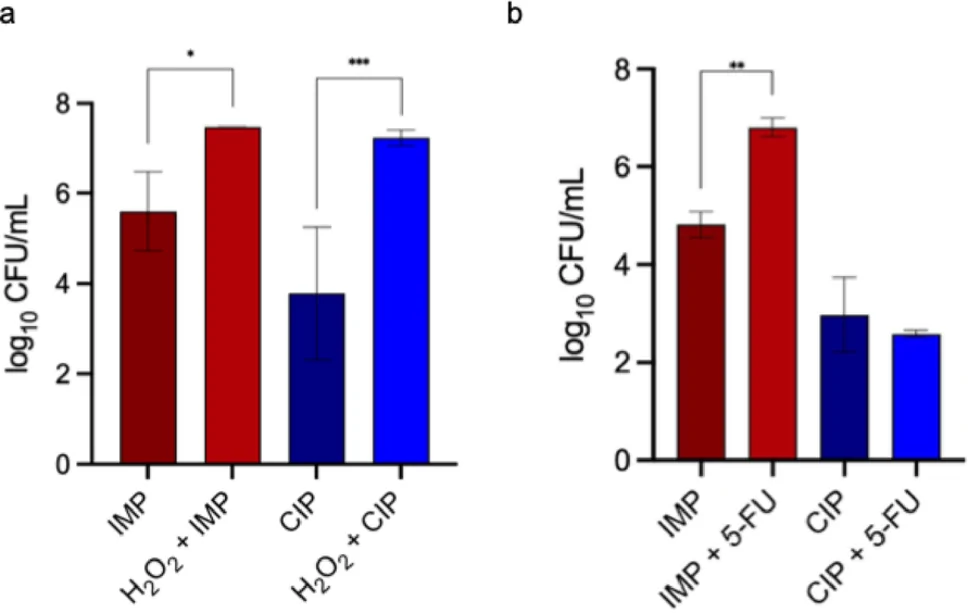
**a** Persister cells counts following treatment with a sub-lethal dose of H_2_O_2_. *P. aeruginosa* PA14 grown overnight (~ 18 h) was exposed to 0.4% H_2_O_2_ for 1 h. Cells were then exposed to imipenem or ciprofloxacin at 30 × MIC (60 or 3.75 μg/mL, respectively) for 24 h. **b** Persister cell detection after DNA damage induction. Overnight (~ 18 h) PA14 cells grown in LB were exposed to 5-FU at 256 µg/mL for 4 h, followed by treatment with 30 × MIC of imipenem (60 μg/mL) or ciprofloxacin (3.75 μg/mL) for 24 h. Cells were washed and plated out on LB agar plates. *IMP* imipenem, *CIP* ciprofloxacin. All experiments were conducted in at least three independent biological replicates. The Shapiro–Wilk test was applied to assess data normality, followed by a Student's t-test to compare two groups. *p < 0.05; **p < 0.01; ***p < 0.001

In addition to damaging bacterial components (da Cruz Nizer et al. 2021), ROS have been implicated in persistence by activating oxidative stress response mechanisms (Grant and Hung 2013). First, to investigate whether H_2_O_2_ stress alters the numbers of persistent cells in *P. aeruginosa* PA14, cells grown overnight were exposed to a sub-lethal concentration of 0.4% H_2_O_2_ for 1 h. The exposure time was optimized using a preliminary killing curve (Supplementary Figure S3), in which cells were treated for 15, 30, 60, and 120 min. A 15- and 30-min exposure did not result in bacterial killing, whereas 1 h and 2 h treatments reduced bacterial viability, with 2 h showing enhanced effect. Therefore, 1 h was selected as the shortest exposure time that produced an oxidative stress effect without considerable killing. Cells were then exposed to imipenem or ciprofloxacin at 30 × MIC (60 or 3.75 μg/mL, respectively) for 24 h. Overall, pre-exposure to H_2_O_2_ prior to antibiotic treatment increased the number of persister cells. Following imipenem treatment, control cells (i.e., PA14 not exposed to H_2_O_2_) showed a log_10_ CFU/mL of 5.9, whereas cells pre-exposed to H_2_O_2_ exhibited higher survival, with a log_10_ CFU/mL of 7.5. For ciprofloxacin, cells not exposed to H_2_O_2_ had a log_10_ CFU/mL of 3.8, while in H_2_O_2_-treated cells this value reached 7.2, representing a more than 3-log_10_ increase in survival (Fig. 5a). These results indicate that exposure to H_2_O_2_ increases the amount of persister cell detection after exposure to imipenem and ciprofloxacin.

We then evaluated the effect of DNA damage on persistence. The SOS response is a well-characterized mechanism involved in persistence (Podlesek and Žgur Bertok 2020). DNA-damaging agents such as 5-FU can trigger the expression of DNA repair and persistence-related genes, such as *recA* (Zhang et al. 2024). Based on previous findings in *E. coli* showing that sub-inhibitory concentrations of 5-FU upregulate DNA repair pathways (Zhang et al. 2024), we investigated the role of DNA damage and SOS response in persistence in *P. aeruginosa* PA14. For this, overnight PA14 cells grown in LB were exposed to a sub-lethal concentration of 5-FU (256 µg/mL) for 4 h, followed by treatment with 30 × MIC of imipenem (60 μg/mL) or ciprofloxacin (3.75 μg/mL) for 24 h. Our results revealed that pretreatment with 5-FU led to a 2-log_10_ increase in imipenem-persistent cells, with log_10_ CFU/mL values rising from 4.8 in the control (no 5-FU pre-treatment) to 6.8. On the other hand, no difference in persistence was obtained for ciprofloxacin (Fig. 5b). These results suggest that DNA damage can enhance imipenem-persister cell detection under the conditions tested, whereas no additional effect was observed for ciprofloxacin.

### Imipenem- and ciprofloxacin-persistent *P. aeruginosa *PA14 cells present distinct yet reduced traits associated with virulence

#### Pigment production

**Fig. 6:**
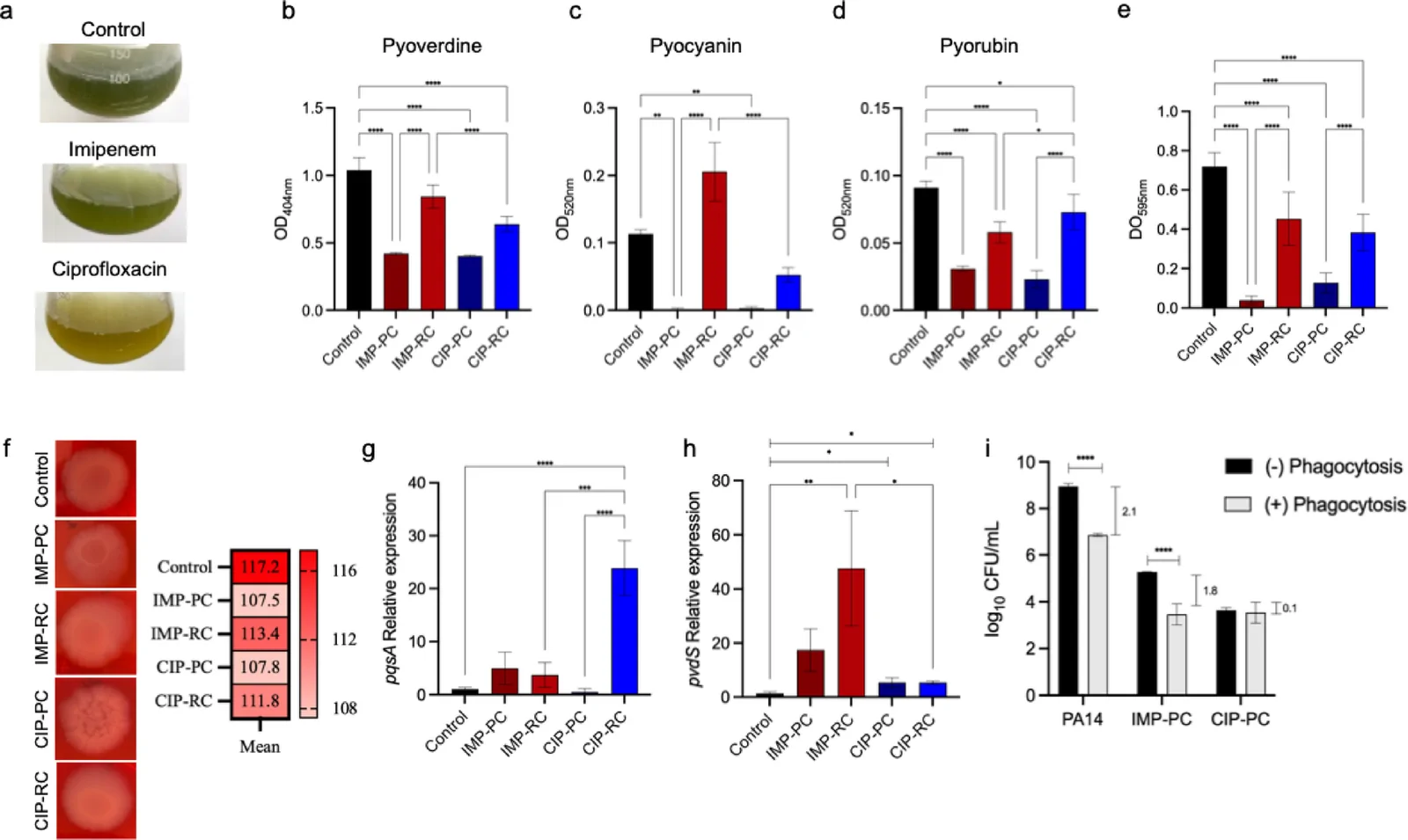
Virulence profile of *P. aeruginosa* PA14 persister and recovered cells. **a**–**d** Pigment production in PA14 persistent and recovered cells. Pyoverdine was quantified by collecting the supernatants from 24 h cultures and measuring the OD at 404 nm. Pyocyanin and pyorubin production were measured using the chloroform method in the organic and aqueous phases, respectively, at 520 nm. **e** Biofilm biomass quantification. PA14 persister or recovered cells were grown in LB for 24 h under static conditions to allow biofilm formation. Biofilms were washed and stained with 0.1% crystal violet solution. OD at 595 nm was measured. **f** Congo red assay. Aliquots of 10 µL of persister and recovered cells were spotted onto LB agar plates supplemented with 40 mg/L Congo red and incubated for 24 h at 37 °C. Colony color intensity was analyzed by ImageJ. Gene expression of **g** *pqsA* and **h** *pvdS* in PA14 persistent and recovered cells. PA14 persister cells were grown for 8 h in LB supplemented with 30 × MIC of imipenem (60 µg/mL) or ciprofloxacin (3.75 µg/mL), and recovered cells were grown in LB for 24 h. Control cells were also grown for 8 h. (i) Susceptibility of *P. aeruginosa* PA14 to phagocytosis. PA14 overnight cells (~ 18 h) were cultured in LB supplemented with 30 × MIC of imipenem (60 µg/mL) or ciprofloxacin (3.75 µg/mL) for 24 h at 37 °C. Then, the cells were co-incubated with alveolar macrophages AMJ-c11 for 30 min, and gentamicin was added to eliminate extracellular bacteria. Macrophages were lysed with 0.1% Triton X-100 to release intracellular bacteria, and the recovered bacteria were plated out in LB agar plates. IMP-PC: imipenem-persistent cells; CIP-PC: ciprofloxacin-persistent cells; CIP-RC: cells that recovered from ciprofloxacin treatment; IMP-RC: cells that recovered from imipenem treatment. All experiments were conducted in at least three independent biological replicates. The Shapiro–Wilk test was applied to assess data normality, followed by One-Way ANOVA. *p < 0.05; **p < 0.01; ***p < 0.001; ****p < 0.0001

*P. aeruginosa* pigments are associated with oxidative stress response and play a significant role in virulence and resistance to antimicrobial agents (da Cruz Nizer et al. 2021). Additionally, the results obtained in the REPTIS method (Fig. 2c) indicated the formation of a brownish color on the replica plates for ciprofloxacin-recovered cells (Fig. 6a). This could indicate pyorubin production. We then analyzed pigment production in imipenem- and ciprofloxacin-persistent cells grown in LB supplemented with 30 × MIC of imipenem (60 μg/mL) or ciprofloxacin (3.75 μg/mL) for 24 h. Recovered cells grown in LB for 24 h were also included (Fig. 6b-d). Pyoverdine was quantified by collecting the culture supernatant of 24 h cultures and measuring the OD at 404 nm. Pyocyanin and pyorubin production were measured using the chloroform method in the organic and aqueous phases, respectively, at 520 nm. Both persistent cell populations exhibited a marked reduction in pyoverdine levels (OD_404nm_ of approximately 0.4) compared to the control (OD_404nm_ of approximately 1) (Fig. 6b). Ciprofloxacin-recovered cells also showed reduced pyoverdine production (OD_404nm_ of approximately 0.64) relative to the control and imipenem-recovered cells (OD_404nm_ of approximately 1 and 0.84, respectively) (Fig. 6b). Pyocyanin levels were also diminished in persister cells, and ciprofloxacin-recovered cells produced less pigment (OD_520nm_ of 0.05) than the control (OD_520nm_ of 0.11). In contrast, imipenem-recovered cells displayed enhanced pyocyanin production (OD_520nm_ of 0.2) (Fig. 6c), suggesting that these surviving cells may have experienced stress that stimulates pyocyanin production. Pyorubin production was reduced in persister cells, with imipenem treatment resulting in a significant decrease (OD_520nm_ of 0.03) compared with the control (OD_520nm_ of 0.09) and ciprofloxacin-treated cells (OD_520nm_ of 0.02). Notably, pyorubin levels in ciprofloxacin-persistent cells were more than threefold lower than in ciprofloxacin-recovered cells (0.02 to 0.07, respectively) (Fig. 6d). Overall, the production of all three pigments increased in the recovered cells compared to the persistent populations.

#### Biofilm formation analysis

Biofilm formation by imipenem- and ciprofloxacin-persistent cells, as well as their respective recovered cells, was evaluated using the crystal violet assay, with OD measurement at 595 nm. Persistent cells exhibited significantly reduced OD_595nm_ values (0.04 and 0.12 for imipenem and ciprofloxacin, respectively), demonstrating a marked decrease in biofilm formation. On the other hand, the recovered cells showed increased biofilm formation compared to their respective persistent populations (Fig. 6e). Imipenem-recovered cells showed average OD values of 0.45, while the ciprofloxacin-recovered cells reached an OD_595nm_ of 0.38. Although these populations did not reach the levels observed in the control condition (OD_595nm_ of 0.72), the data indicate a significant recovery in biofilm-forming ability following the removal of antibiotic stress.

Next, to evaluate whether reduced biofilm formation in imipenem- and ciprofloxacin-persister cells was associated with decreased production of matrix components such as Pel, we performed a Congo red binding assay to qualitatively assess extracellular matrix production (Lee et al. 2016; Harika et al. 2020). Aliquots of 10 µL of persister and recovered cells were spotted onto LB agar plates supplemented with 40 mg/L Congo red and incubated for 24 h at 37 °C. Colony color intensity was analyzed by ImageJ, and the mean values were plotted as a heat map in GraphPad Prism (Fig. 6f). Consistent with the crystal violet staining results (Fig. 6e), imipenem- and ciprofloxacin-persister cells showed reduced Congo red staining intensity (107.5 and 107.8, respectively) compared to the control (117.2) and their recovered counterparts (113.4 and 111.8, respectively) (Fig. 6f), suggesting that persister cells produce less biofilm biomass, potentially due to decreased production of the extracellular matrix and its components, such as Pel.

### Differential regulation of *pqsA* and *pvdS* genes in persistent and recovered cells

To build on the previous results that persistent cells exhibit reduced pigment production and biofilm formation and that recovered cells present an altered phenotype compared to the control, we conducted a gene expression analysis of the quorum-sensing (QS) genes *pqsA* and pyoverdine (*pvdS*) (Fig. 6g-h). For *pqsA*, imipenem-persistent cells showed elevated expression (4.9) compared to both the control (0.9) and recovered cells (3.7). In contrast, ciprofloxacin-treated cells displayed the highest *pqsA* expression in recovered cells (23.8), whereas expression remained low in the control (0.9) and persistent cells (0.53) (Fig. 6g). This indicates that *pqsA* is induced during persistence in response to imipenem but reaches its highest levels during recovery following ciprofloxacin exposure.

Lastly, ciprofloxacin-persistent and recovered cells showed increased *pvdS* expression (5.4 and 5.3, respectively) compared to the control. On the other hand, *pvdS* expression was enhanced in imipenem-recovered cells (47.6) compared to persistent (17.4) and control cells (Fig. 6h), suggesting that this gene is strongly induced during the recovery phase following beta-lactam treatment.

### Persister cell responses to innate immune challenges reveal differential susceptibility to H_2_O_2_ and phagocytosis

H_2_O_2_ is an important component of the immune system that exerts antimicrobial activity against invading pathogens. We then conducted MIC analysis to evaluate the susceptibility of imipenem or ciprofloxacin-persistent and recovered cells to H_2_O_2_. Imipenem-persistent and recovered cells showed increased susceptibility to H_2_O_2_ (MIC of 0.0098%) compared to the control (MIC of 0.0391%). On the other hand, ciprofloxacin-persistent and recovered cells presented MICs of 0.0391% and 0.019%, respectively, which are similar to the parental strain for the tested antimicrobial agents. For reference, only changes greater than twofold in MIC values are considered meaningful (Mouton et al. 2018b, a; Kadeřábková et al. 2024).

Overall, the previous findings support the conclusion that persistent and recovered cells present an altered virulence profile, depending on the antibiotic to which they were exposed. To assess whether persister cells can withstand host immune clearance, we examined their survival after phagocytosis by alveolar macrophages. For this, *P. aeruginosa* recovered after 30 min of phagocytosis was plated to quantify bacterial uptake (Fig. 6i). Figure 6i shows that a minor fraction of control or imipenem-treated cells is phagocytosed. Specifically, PA14 decreased from 8.9 to 6.8 log_10_ CFU/mL (Δ of 2.1), while imipenem-persistent cells decreased from 5.2 to 3.4 log_10_ CFU/mL (Δ of 1.8). On the other hand, no significant reduction in CFU was observed in the ciprofloxacin-persistent group, with log_10_ CFU/mL values of 3.6 and 3.5 in the non-phagocytosed and phagocytosed groups, respectively, indicating that all the ciprofloxacin-persistent cells were phagocytosed by macrophages. This suggests that ciprofloxacin persister cells are promptly phagocytosed by macrophages, in contrast to imipenem persister cells.

### Phagocytosis reduces persistence in antibiotic-exposed *P. aeruginosa*

**Fig. 7:**
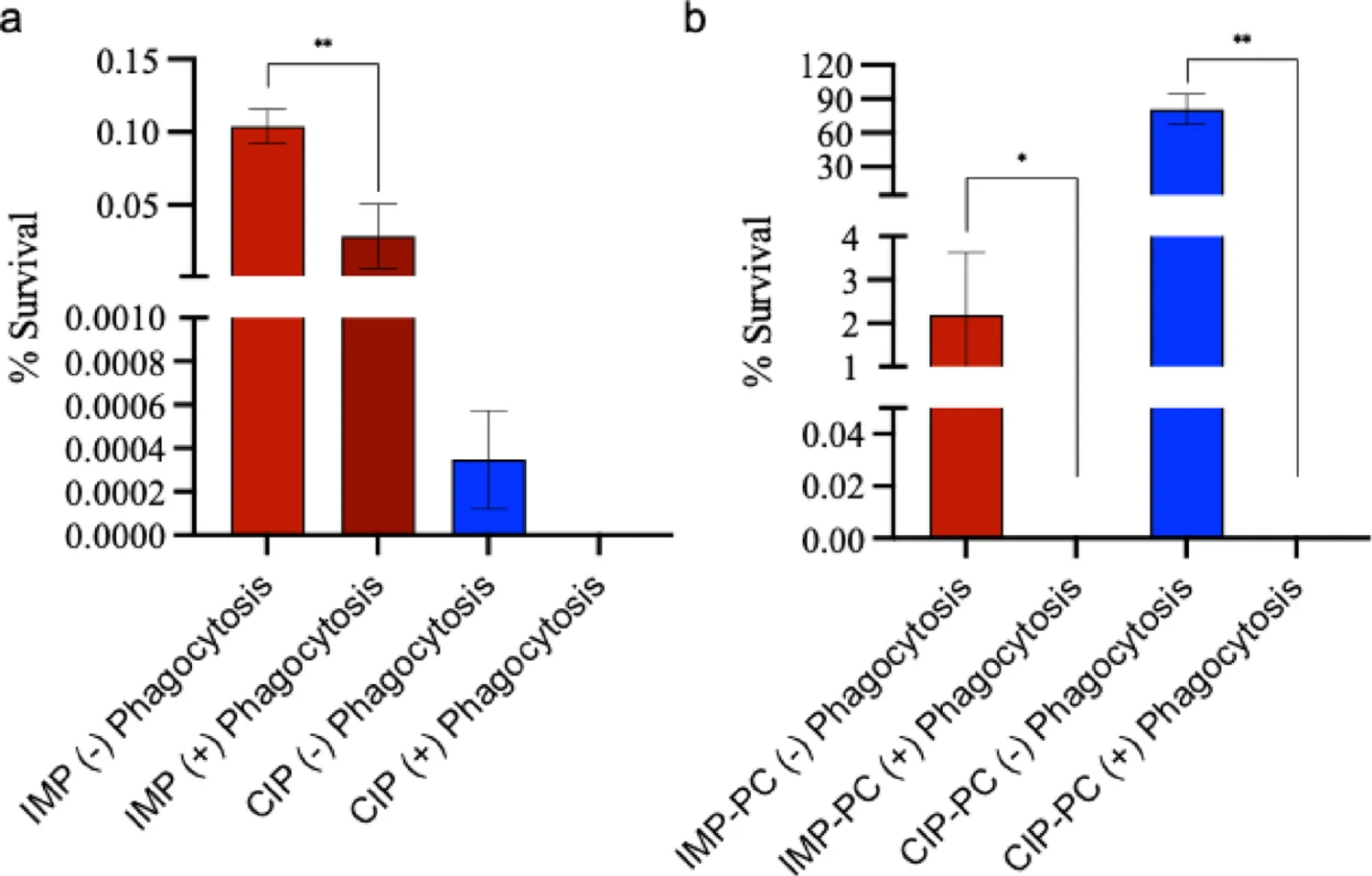
Intracellular survival of *P. aeruginosa* PA14 persister cells following phagocytosis by alveolar macrophages. **a** PA14 and **b** PA14 persister cells. Planktonic *P. aeruginosa* cells grown overnight (~ 18 h) in LB or LB supplemented with 30 × MIC of imipenem (60 µg/mL) or ciprofloxacin (3.75 µg/mL) were co-incubated with alveolar macrophages AMJ-c11 for 30 min, and gentamicin was added to eliminate extracellular bacteria. Macrophages were lysed with 0.1% of Triton X-100 to release intracellular bacteria, and the recovered bacteria were either immediately plated (T_0_) or exposed to 30 × MIC of imipenem (60 µg/mL) or ciprofloxacin (3.75 µg/mL) for 24 h (T_24_) to assess persister cell formation. All experiments were conducted in at least three independent biological replicates. The Shapiro–Wilk test was applied to assess data normality, followed by a Student's t-test to compare two groups. *p < 0.05; **p < 0.01

The innate immune system represents the first line of defense against invading pathogens, such as *P. aeruginosa,* and has been shown to alter the antimicrobial susceptibility of these pathogens (Handel et al. 2009). To investigate whether phagocytosis affects persistence, we performed a phagocytosis assay using AMJ2-c11 alveolar macrophages. Following phagocytosis for 30 min, extracellular bacteria were killed with gentamicin, and intracellular bacteria were recovered from macrophages and subsequently exposed to 30 × MIC of imipenem (60 µg/mL) or ciprofloxacin (3.75 µg/mL) for 24 h. Survival was calculated as the percentage of cells remaining after antibiotic exposure, with the number of bacteria recovered after phagocytosis set to 100%. As shown in Fig. 7a, the number of persistent cells was smaller in PA14 cells that had undergone phagocytosis. After exposure to imipenem, phagocytosed cells showed a survival rate of 0.03% compared with 0.1% in the non-phagocytosed control. An even more pronounced effect was observed with ciprofloxacin, where no cells were detected after antibiotic exposure following phagocytosis, compared with 0.0003% in the non-phagocytosed control.

To assess whether the effect observed in PA14 was strain-specific, persister cell counts after phagocytosis were evaluated in clinical isolates 12-0048 and 16-0040 (Supplementary Figure S4). Similar to PA14, the clinical isolates showed reduced rates of persister cells when phagocytosed cells were treated with 30 × MIC of imipenem compared to non-phagocytosed controls (Supplementary Figure S4), suggesting that phagocytosis reduces the number of persister cells and increases antibiotic susceptibility.

Building on previous findings that phagocytosis by AMJ2-c11 alveolar macrophages reduces imipenem- and ciprofloxacin-persister cell numbers in *P. aeruginosa* PA14 and clinical isolates, and because imipenem or ciprofloxacin persister cells exhibit distinct virulence profiles, we asked whether their response to phagocytosis would also differ. Then, we evaluated whether PA14 persister cells (i.e., planktonic PA14 cells previously exposed to 30 × MIC of imipenem or ciprofloxacin for 24 h) could retain their persistent phenotype upon antibiotic exposure after phagocytosis. After phagocytosis, PA14 cells were recovered, exposed to 30 × MIC of imipenem or ciprofloxacin for 24 h, and the percentage survival was calculated. Persistent PA14 cells that underwent phagocytosis did not survive subsequent exposure to imipenem and ciprofloxacin, as no viable cells were detected after 24 h of incubation with either antibiotic (Fig. 7b). These findings suggest that although imipenem- and ciprofloxacin-persistent cells survive phagocytosis by alveolar macrophages, this interaction alters their physiology, restoring susceptibility to antibiotic treatment, suggesting that intracellular conditions may disrupt persistence mechanisms.

## Discussion

Nearly 80 years ago, researchers observed that antibiotics, despite being effective against susceptible bacterial strains, failed to eradicate bacterial populations (Hobby et al. 1942; Bigger 1944). Today, the recurrence and chronicity of bacterial infections are recognized as being driven not only by antimicrobial resistance but also by bacterial persistence (La Rosa et al. 2025). While the detection of persister cells upon antibiotic exposure has been described in *P. aeruginosa* (Patel et al. 2022; Pont et al. 2025) and other bacterial species (Keren et al. 2011; Ovsepian et al. 2020; Umetani et al. 2025), several aspects of their biology remain poorly understood. In this study, we investigated the phenotype of persister cells in *P. aeruginosa* following exposure to imipenem and ciprofloxacin, two clinically relevant antibiotics in the management of *P. aeruginosa* infections (Soares et al. 2020). Using different experimental set-ups, including persister detection in solid and liquid media, planktonic and surface-attached cells, and an in vitro phagocytosis model, we characterized the virulence of persister cells. Notably, we show that oxidative stress and DNA damage increase the levels of persister cells. Furthermore, we demonstrate that, although these cells exhibit reduced virulence-associated phenotypes, imipenem- and ciprofloxacin-recovered cells present differential virulence patterns, providing new insights into the phenotypic state of these *P. aeruginosa* populations.

Several well-established criteria must be met for cells to be classified as persistent: (i) persister cells are non-replicative; (ii) they survive exposure to high concentrations of bactericidal antibiotics without acquiring genetic resistance (Figs. 1a-d, Table 2); (iii) they exhibit a biphasic killing pattern (Fig. 2d); (iv) after antibiotic removal, their progeny remains as susceptible as the parental strain (Fig. 2, Table 2); and (v) the proportion of persisters remains relatively constant even when antibiotic concentrations increase, as long as they are above the MIC (Fig. 2a-b) (Balaban et al. 2019; Sett et al. 2024). Overall, these criteria were met in our study, confirming that the surviving subpopulations observed after treatment with imipenem and ciprofloxacin are indeed persisters (Figs. 1 and 2, and Table 2). Despite this, flow cytometry parameters (Fig. 3) alone are insufficient to definitively identify persister cells, as they do not capture key features such as regrowth after antibiotic removal or the absence of heritable resistance. Therefore, flow cytometry data should be interpreted alongside classical phenotypic criteria.

While there is no universally standardized concentration for persistence assays, they must use antibiotic concentrations well above the MIC to ensure rapid killing of susceptible cells and to prevent the survival of resistant bacteria (Balaban et al. 2019). In the literature, a broad range of concentrations has been used depending on the bacterial species, antibiotic, and experimental design. For example, studies have used 5 × MIC of gentamicin, ciprofloxacin, and ceftazidime (Patel et al. 2022); 10 × MIC of cefotaxime, ofloxacin, and tobramycin (Keren et al. 2004); 12.5 × MIC of ampicillin and 32 × MIC of ciprofloxacin (Umetani et al. 2025); and even up to 200 × MIC of ciprofloxacin (Ovsepian et al. 2020). In our study, we detected persister cells after treatment with 30 × MIC of imipenem and ciprofloxacin. Additionally, longer antibiotic exposure times have been shown to increase the detectable fraction of persister cells. For instance, Dewachter et al. (2021) reported that exposure to ofloxacin for 32 h resulted in approximately 25% of persister cells (Dewachter et al. 2021).

In addition to the antibiotic concentration, the physiological state of the bacterial population is a key determinant of persistence. To control for this variable, we used stationary cells of *P. aeruginosa* grown overnight in LB medium. This approach not only excluded differences in drug tolerance associated with different growth phases (Mulcahy et al. 2010) but also ensured consistency with prior persistence studies. In this context, several reports have demonstrated that stationary-phase populations harbor a significantly higher proportion of persisters compared to exponentially growing cells (Spoering and Lewis 2001; Keren et al. 2004, 2011; Conlon et al. 2016; Umetani et al. 2025). This can be attributed to the high density of stationary-phase populations of *P. aeruginosa* (Spoering and Lewis 2001), decreased ATP levels in *S. aureus* (Conlon et al. 2016), and morphological changes in cell shape of *E. coli* (Umetani et al. 2025).

The biphasic killing curve characteristic of persister cells reflects the phenotypic heterogeneity of these bacterial populations (Balaban et al. 2004), in which rapid killing of susceptible cells is followed by a slower or no-killing phase during which persisters remain (Wilmaerts et al. 2019). This heterogeneity was further supported by a recent study that showed diverse survival behaviors among individual bacteria within a population (Umetani et al. 2025). Using single-cell analysis of *E. coli*, Umetani et al. (2025) revealed heterogeneous behaviors upon ampicillin exposure, in which some cells arrested division during treatment and resumed growth only after antibiotic removal, while others continued growing with abnormal, L-like shapes. These L-like cells exhibited unusual motility and fragmentation yet were still able to regenerate rod-shaped progeny (Umetani et al. 2025). In accordance with our findings, Patel and collaborators (2022) obtained the biphasic killing curves for three isolates of *P. aeruginosa* treated with 5 × MIC of gentamicin, ciprofloxacin, and ceftazidime and reported that the shape of the curve varied depending on the isolate and antibiotic used, with ciprofloxacin causing the greatest reduction in viable cells (Patel et al. 2022).

The presence of persister cells is a major contributor to the resilience of bacterial biofilms and plays a key role in the recalcitrance of biofilm-related infections (Lewis 2008; Yan and Bassler 2019). We showed a significantly higher level of persister cells in surface-attached populations compared to non-attached (planktonic) cells (Fig. 2f), reinforcing that biofilm-associated cells are more resilient to antibiotic killing. In accordance, a recent study showed that cell adhesion favors persistence in Uropathogenic *E. coli* (Liao et al. 2024). In this context, the authors showed that adhesion to surfaces is accompanied by an increase in cyclic-di-GMP (c-di-GMP) (Liao et al. 2024), a second messenger essential for, among others, biofilm formation and resistance (Gupta et al. 2014; Strempel et al. 2017; Liu et al. 2022). This increase led to the initiation of regulatory pathways that favor bacterial dormancy and antibiotic tolerance (Liao et al. 2024). Moreover, c-di-GMP also led to upregulated expression of HipH, a DNase capable of introducing DNA double-strand breaks. Interestingly, c-di-GMP simultaneously counteracts the genotoxic activity of HipH, acting as an antitoxin while promoting persistence, thereby highlighting its dual role in coordinating stress adaptation and survival within biofilms (Liao et al. 2024).

The extracellular polymeric substance (EPS) matrix and its components represent a physical and chemical barrier that impedes antibiotic penetration (Harimawan and Ting 2016; Hu et al. 2019) or interacts with antimicrobials to reduce their local concentration (Colvin et al. 2011; Billings et al. 2013; da Cruz Nizer et al. 2024b), which can lead to antimicrobial resistance and persistence. Indeed, Patel et al. (2022) showed that antibiotic penetration into biofilms and biofilm structure directly influence bacterial survival (Patel et al. 2022). However, persister survival appears to depend on more than matrix protection alone. Biofilms formed by a mutant lacking both exopolysaccharides Psl and Pel (∆*pelA*∆*pslBCD*) were nearly eradicated by tobramycin and ciprofloxacin, with only a small subpopulation of persister cells remaining (Yang et al. 2011). Since biofilm cells in our study were washed prior to antibiotic treatment, the increased levels of persisters are likely influenced not only by the presence of cell-associated Pel (Jennings et al. 2015; da Cruz Nizer et al. 2024b) but also by the low metabolic state of surface-attached populations (Spoering and Lewis 2001). Consistent with this, Spoering and Lewis (2001) showed that even after EPS removal, *P. aeruginosa* biofilms retained tolerance to tobramycin due to slow growth (Spoering and Lewis 2001).

In accordance with previous studies suggesting that persisters present a dormant, low-metabolic state to withstand antibiotic stress (Balaban et al. 2019; Patel et al. 2022), we found that persister cells exhibit overall reduced virulence-associated traits in vitro. Accordingly, *P. aeruginosa* and *S. aureus* persister cells also exhibit delayed virulence and elicit an attenuated immune response in in vivo models (Mina and Marques 2016; Hastings et al. 2023b). Therefore, since exposure to high antibiotic concentrations causes substantial metabolic suppression, the reduced pigment production and biofilm formation observed in this study may reflect a general reduction in metabolic activity rather than a specific reprogramming of virulence pathways. Yet, this reduced virulence may contribute to adaptive strategies, such as energy conservation or immune evasion. Supporting this idea, *Acinetobacter baumannii* exposed to 50 × MIC of ceftazidime (Alkasir et al. 2018), *M. tuberculosis* exposed to D-cycloserine (Keren et al. 2011), and *Salmonella enterica* exposed to 100 × MIC of ciprofloxacin and ceftazidime (Mattiello et al. 2023) exhibited downregulation of genes associated with energy metabolism, consistent with a reduced metabolic state.

We also investigated the mechanisms contributing to persistence in PA14 exposed to imipenem and ciprofloxacin and observed that exposure to H_2_O_2_, an important source of oxidative stress in host environments (da Cruz Nizer et al. 2021, 2024a), led to a marked increase in persister cell numbers. Similar observations have been reported in *E. coli*, where sublethal concentrations of paraquat or salicylate enhanced persistence by generating reactive oxygen species (ROS) and subsequent activation of stress-response pathways (Wu et al. 2012; Wang et al. 2017). In addition to oxidative stress, DNA damage is increasingly recognized as a key factor contributing to bacterial persistence. To investigate its potential role in imipenem and ciprofloxacin persistence, we used the genotoxic agent 5-FU. Indeed, co-treatment with 5-FU for 4 h significantly increased persister levels during imipenem exposure but not during ciprofloxacin exposure. The lack of effect of 5-FU pretreatment on ciprofloxacin persistence could be due to ciprofloxacin acting by damaging DNA and activating the SOS response (Cirz et al. 2007; Dörr et al. 2009). Therefore, we hypothesize that under our experimental conditions, ciprofloxacin treatment may already maximize the activation of DNA damage-associated stress responses, thereby limiting any additional effect of DNA damage on persister phenotype. The 4 h exposure time was selected based on previous reports demonstrating that prolonged induction of the SOS response enhances persistence. Specifically, treatment with mitomycin C for 4 h resulted in a markedly greater increase in fluoroquinolone persistence than 2 h of exposure (Dörr et al. 2009). As 5-FU also induces DNA damage (Zhang et al. 2024) and triggers the SOS response (Dörr et al. 2009), a 4 h exposure was used to ensure adequate activation of the pathway. In *E. coli*, 5-FU disrupts DNA metabolism by inhibiting thymidylate synthase, leading to activation of the SOS response via *recA*- and *lexA*-dependent pathways (Oda 1987; Zhang et al. 2024). Based on these findings, we hypothesized that 5-FU could modulate *P. aeruginosa* persistence via an SOS-mediated mechanism. Interestingly, the involvement of the SOS response in persistence appears to be species- and context-dependent, as supported by findings reported here and by other researchers (Dörr et al. 2009; Ovsepian et al. 2020).

Interestingly, although they recover the phenotype compared to persister cells, we show that recovered populations present altered phenotypes after the removal of antibiotic stress. Notably, imipenem-recovered populations produced elevated levels of pyocyanin (Fig. 6c), a redox-active virulence factor produced by *P. aeruginosa* that contributes to oxidative stress (Muller 2002; da Cruz Nizer et al. 2021), host tissue damage (Hall et al. 2016), and immune modulation (Lew et al. 2025). The reduced production of pigments and biofilm observed in persister-enriched populations is consistent with the reduced metabolic activity associated with bacterial dormancy. Moreover, the differential regulation of *pqsA* and *pvdS* under imipenem and ciprofloxacin exposure suggests that distinct antibiotics may differentially influence quorum-sensing and virulence-regulatory pathways.

Furthermore, ciprofloxacin-recovered cells displayed elevated expression of persistence-associated genes (*recA, relA, spoT, lon*, and *higB*), suggesting that antibiotic stress not only selects for persisters but also programs the recovered population with a transcriptional profile that may increase tolerance to subsequent stresses. Notably, no amplification of stringent response-associated genes (*relA*, *spoT*, and *lon*) was detected in persister cells collected at 8 h. As this time point corresponds to the early stages of persister detection, this finding likely reflects reduced or highly heterogeneous transcriptional activity among a heterogeneous population harboring susceptible and persistent cells. Importantly, transcriptional responses to antibiotics are highly time-dependent, with distinct gene expression observed across different exposure times, as demonstrated in *M. tuberculosis*, where antibiotic-specific transcriptional profiles vary significantly within the first hours of treatment (Poonawala et al. 2024). Furthermore, it is also possible that expression levels were below the detection limit of our experimental conditions. Upon recovery, however, the marked upregulation of *relA*, *spoT*, and *lon* suggests a rapid reactivation of the stringent response, potentially facilitating adaptation to post-antibiotic conditions and enhancing survival under renewed stress. Supporting this, a study examining *P. aeruginosa* biofilms exposed to ciprofloxacin documented upregulation of stringent response regulators (*relA*, *spoT*, and *lon*) and toxin-antitoxin components such as *higBA* within 1 h of treatment (Patel et al. 2022). However, studies of recovered populations remain scarce.

Building on the results from the reduced virulence of persister cells, we showed that *P. aeruginosa* persister cells were not detected among cells that underwent phagocytosis by alveolar macrophages. This suggests that the intracellular environment imposes stress conditions incompatible with the activation or maintenance of persistence mechanisms. One possibility is that phagocytosed cells enter a dormant state but lack the time or metabolic resources to recover before facing a second antibiotic exposure. In parallel, oxidative stress agents generated by macrophages, such as ROS and reactive chlorine species (RCS), can damage essential cellular systems (da Cruz Nizer et al. 2020, 2021), potentially impairing the stress responses required for persister survival. Notably, the survival of persistent cells after phagocytosis supports the idea that persisters are more tolerant not only to antibiotics but also to host-mediated killing. This is consistent with previous findings showing that *P. aeruginosa* persister cells are engulfed at lower rates by THP-1 macrophages and modulate macrophage polarization toward an M2b-like phenotype, which is more immunosuppressive, before eventually reverting to an M1-polarizing profile as the persisters awaken (Hastings et al. 2023a).

## Conclusion

Our findings demonstrate persistence in *P. aeruginosa* upon exposure to high concentrations of imipenem and ciprofloxacin. Importantly, our results indicate that persistence is not merely a passive, dormant state but rather a dynamic and multifaceted survival strategy in response to antibiotic exposure, depending on the nature of the stress cells face. While previous studies have primarily focused on persister cell phenotypes and its underlying mechanisms, to our knowledge, this is the first work to provide a detailed phenotypic characterization of recovered populations. These insights broaden our understanding of how *P. aeruginosa* adapts not only to antibiotic pressure but also to subsequent host-derived stresses and post-antibiotic exposure. However, some limitations of this study include assessing persistence using in vitro models, which do not fully recapitulate the complexity of in vivo environments, where factors such as host immune responses, nutrient gradients, and tissue architecture can significantly influence bacterial physiology and antibiotic tolerance. Furthermore, antibiotic activity was assessed under specific culture conditions known to enhance persister cell detection (e.g., media composition and growth phase), which are known to influence drug efficacy. Future studies using in vivo infection models will be critical to validate the physiological relevance of persister and recovered cells and to guide the development of therapeutic strategies targeting persisters in clinical settings.

## Supplementary Information

Below is the link to the electronic supplementary material.


Supplementary Material 1


## Data Availability

No datasets were generated or analysed during the current study.
