# Supplementary material for: Antibiotic exposure and recovery of persister states influence virulence phenotypes in Pseudomonas aeruginosa
Source: Arch Microbiol. 2026 Jun 16;208(9):437. doi: 10.1007/s00203-026-04995-3 (PMC13272224; doi:10.1007/s00203-026-04995-3)
Supplement: Supplementary file 1 — Supplementary Material 1 [file 203_2026_4995_MOESM1_ESM.docx]

**Antibiotic exposure and recovery of persister states influence virulence phenotypes in** *Pseudomonas aeruginosa*

Waleska Stephanie da Cruz Nizer^ab#^; Carlos Eduardo Dias Igídio^ab^; Estela Mafra Ribeiro^ab^; Allanis Cristiny Oliveira Andrade^ab^; Samantha Neves de Oliveira^ab^; Mauro Martins Teixeira^b^; Daniele da Glória Souza^a^; Caio Tavares Fagundes^ab#^

^a^Host-patogen interaction laboratory, Department of Microbiology, Institute of Biological Sciences, Federal University of Minas Gerais, Belo Horizonte, MG, Brazil.

^b^ Drug Research and Development Center, Institute of Biological Sciences, Federal University of Minas Gerais, Belo Horizonte, MG, Brazil.

^#^Corresponding authors:

da Cruz Nizer, Waleska Stephanie, [waleskaob@gmail.com](mailto:waleskaob@gmail.com)

Fagundes, Caio Tavares, [caio.fagundes@gmail.com](mailto:caio.fagundes@gmail.com) and [ctfagundes@ufmg.br](mailto:ctfagundes@ufmg.br)

Journal Archives of Microbiology

**Figures**

**Supplementary Figure S1** Persister cell detected by the REPTIS method. The optical density at 600 nm (OD_600nm_) of *P. aeruginosa* PA14 grown overnight in LB was adjusted to 0.1 (~ 10^8^ CFU/mL), and cells were plated out on LB agar plates containing 30 × MIC of imipenem (60 µg/mL) or ciprofloxacin (3.75 µg/mL) for 72 h (master plates). The content of the master plates was collected using a saline-soaked sterile swab, transferred to 1 mL of saline, serially diluted, and plated on LB agar plates without antibiotics. All experiments were conducted in at least three independent biological replicates.

**Supplementary Figure S2** Persister cells detection in non-attached and attached populations. Clinical isolate 12-0048 (a) treated with imipenem and ciprofloxacin and isolate 16-0040 (c) treated with imipenem. Biofilms of *P. aeruginosa* were grown on 12-well plates for 24 hours in LB at 37^o^C and static conditions. Non-attached (planktonic) cells were collected and transferred to microtubes, and biofilms (attached cells) were washed with saline solution, and the cells collected by vigorous pipetting. Both cell populations were treated with 30 × MIC of imipenem (60 µg/mL) or ciprofloxacin (3.75 µg/mL) for 4 h, and the CFU/mL was determined. Normality test was performed using GraphPad Prism and data were analyzed by t-test. IMP: imipenem; CIP: ciprofloxacin. All experiments were conducted in at least three independent biological replicates. *p<0.05; **p<0.01.


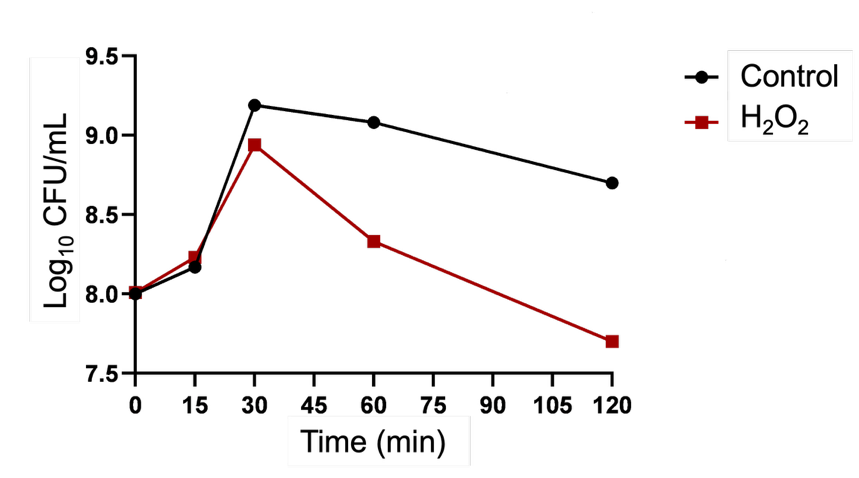


**Supplementary Figure S3** Killing kinetics of H_2_O_2_-exposed *P. aeruginosa* PA14. PA14 cells at 10^8^ CFU/mL were exposed to 0.4% H_2_O_2_ for 15, 30, 60, and 120 min, and CFU/mL was determined. All experiments were conducted in at least three independent biological replicates.

**Supplementary Figure S4** Proportion of persister cells of clinical isolates (a) 12-0048 and (b) 16-0040 after phagocytosis. Planktonic *P. aeruginosa* cells grown overnight in LB or LB supplemented with 30 × MIC of imipenem (60 µg/mL) or ciprofloxacin (3.75 µg/mL) were co-incubated with alveolar macrophages AMJ-c11 for 30 minutes and gentamicin was added to eliminate extracellular bacteria. Macrophages were lysed with 0.1% of Triton X-100 to release intracellular bacteria, and the recovered bacteria were either immediately plated or exposed to 30 × MIC of imipenem (60 µg/mL) or ciprofloxacin (3.75 µg/mL) for 24 hours to assess the number of persister cells. Normality test was performed using GraphPad Prism and data were analyzed by t-test. All experiments were conducted in at least three independent biological replicates. *p<0.05; **p<0.01.
